# *Coxiella burnetii* vascular graft infection

**DOI:** 10.1016/j.idcr.2021.e01230

**Published:** 2021-07-21

**Authors:** Takaaki Kobayashi, Fernando Casado Castillo, Jason H. Barker, Loreen Herwaldt

**Affiliations:** Division of Infectious Diseases, Department of Internal Medicine, University of Iowa Hospitals and Clinics, United States

**Keywords:** *Coxiella burnetii*, Vascular graft infection

## Abstract

Q fever, a zoonotic infection caused by *Coxiella burnetii*, can present with a wide spectrum of clinical manifestations. The organism is typically transmitted from sheep, goats, or cattle to humans via contaminated aerosols. On average, 1–5% of patients with acute Q fever will develop chronic infection months to decades after their primary infections. We report a case of a chronic vascular graft infection due to *Coxiella burnetii* in a 61-year-old man without direct exposure to animals who presented with recurrent fever. Indium-111-labeled white blood cell scan with single-emission positron computed tomography demonstrated findings suggesting a graft infection. *C. burnetii* phase I and phase II IgG antibody titers were > 1:32,768 and polymerase chain reaction performed on the explanted graft was positive for *C. burnetii*. Q fever should be considered in the differential diagnosis of vascular infections in patients who have a pre-existing lesion such as an aneurysm, or vascular prosthesis even in the absence of a history of direct animal exposure.

## Introduction

Q fever is a zoonotic infection caused by *Coxiella burnetii* and can present with a wide spectrum of clinical manifestations. Q fever is typically transmitted from sheep, goats, or cattle to humans via contaminated aerosols. We report a case of a chronic vascular graft infection due to *Coxiella burnetii* in a 61-year-old man without direct exposure to animals who presented with recurrent fever.

## Case

**Fig. 1:**
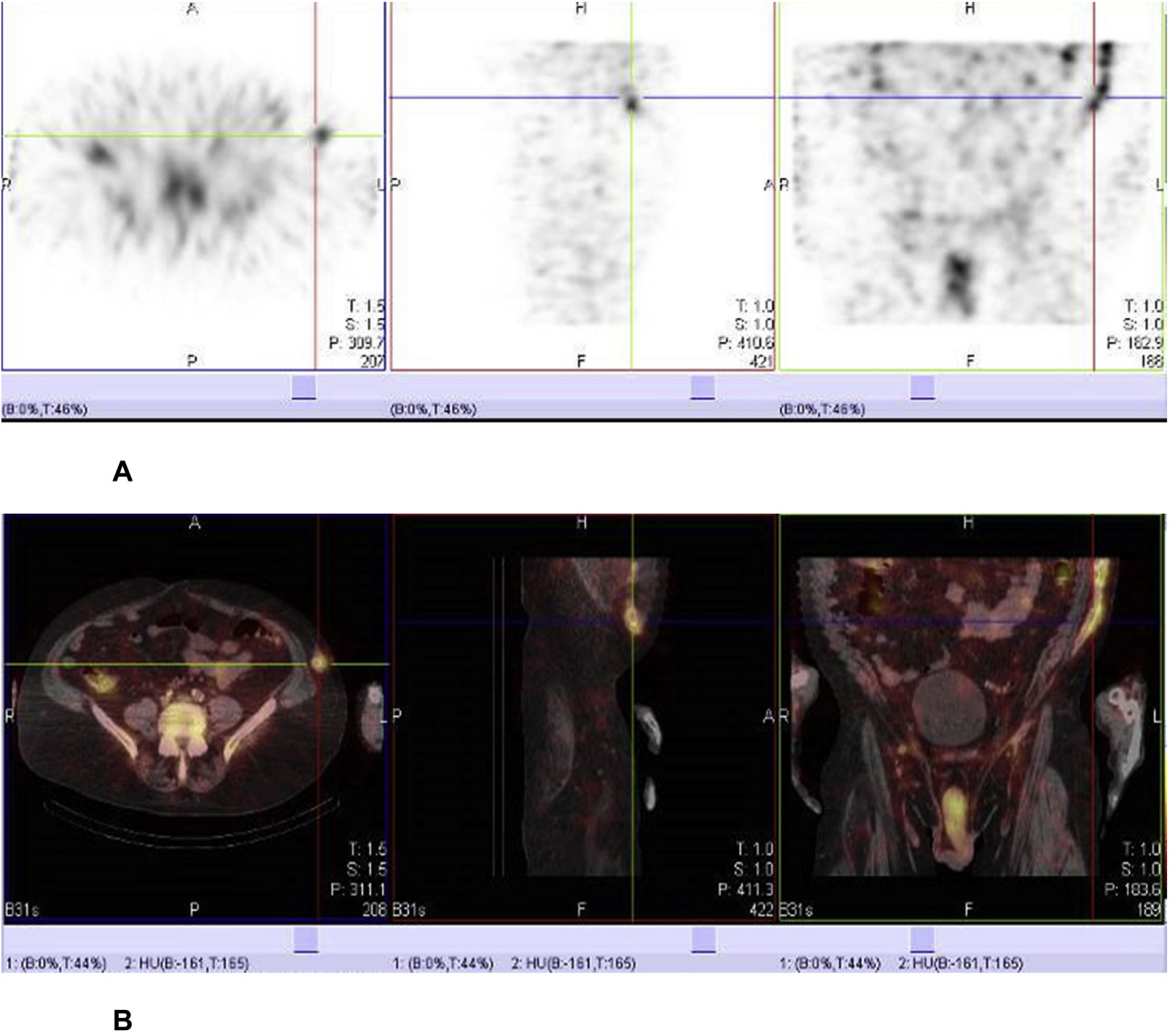
**A.** Multiple cross sections (*right: transverse, middle: sagittal, left: coronal views*) of an Indium-111-labeled white blood cell tagged scan showing focal increased uptake in the axillary-bifemoral graft between the spleen and pelvis suggestive of focal infection. **B.** Multiple cross sections (*right: transverse, middle: sagittal, left: coronal views*) of a single emission positron computed tomography-computed tomography (SPECT-CT) matching an Indium-111 labeled scan (Fig. 1A), showing focal increased uptake in the axillary-bifemoral graft between the spleen and pelvis suggestive of focal infection.

A 61-year-old male presented to his primary care physician (PCP) complaining of intermittent chills, fever, and sweats. The patient had undergone a left axillary-bifemoral bypass graft surgery to repair an abdominal aneurysm 6 years prior. This procedure was complicated by a spinal cord infarction and paraplegia. The results of a complete blood count (CBC), a comprehensive metabolic panel (CMP), a thyroid stimulating hormone, and a urinalysis were within normal limits. Blood cultures were not obtained. His symptoms resolved within a few days and further work up was deferred. However, within 2 weeks, the fevers and sweats recurred. The symptoms continued to recur every 2–3 weeks, and each episode lasted 3–4 days. During the episodes, he had fever as high as 39.8 °C, chills, and sweats. Over the next 3 months, his CBC and CMP remained within normal limits, but his C-reactive protein and erythrocyte sedimentation rate increased from 2.4 mg/dL and 33 mm/Hr, respectively, to 4.8 mg/dL and 39 mm/Hr. Two blood cultures were negative, and a chest radiograph did not find evidence of infection. Three months after symptom onset, an Indium-111-labeled white blood cell scan with single emission positron computed tomography (SPECT-CT) found focal increased radio-tracer uptake in the axillary-bifemoral graft suggestive of focal infection (Fig. 1**A** and **B**).

**Fig. 2:**
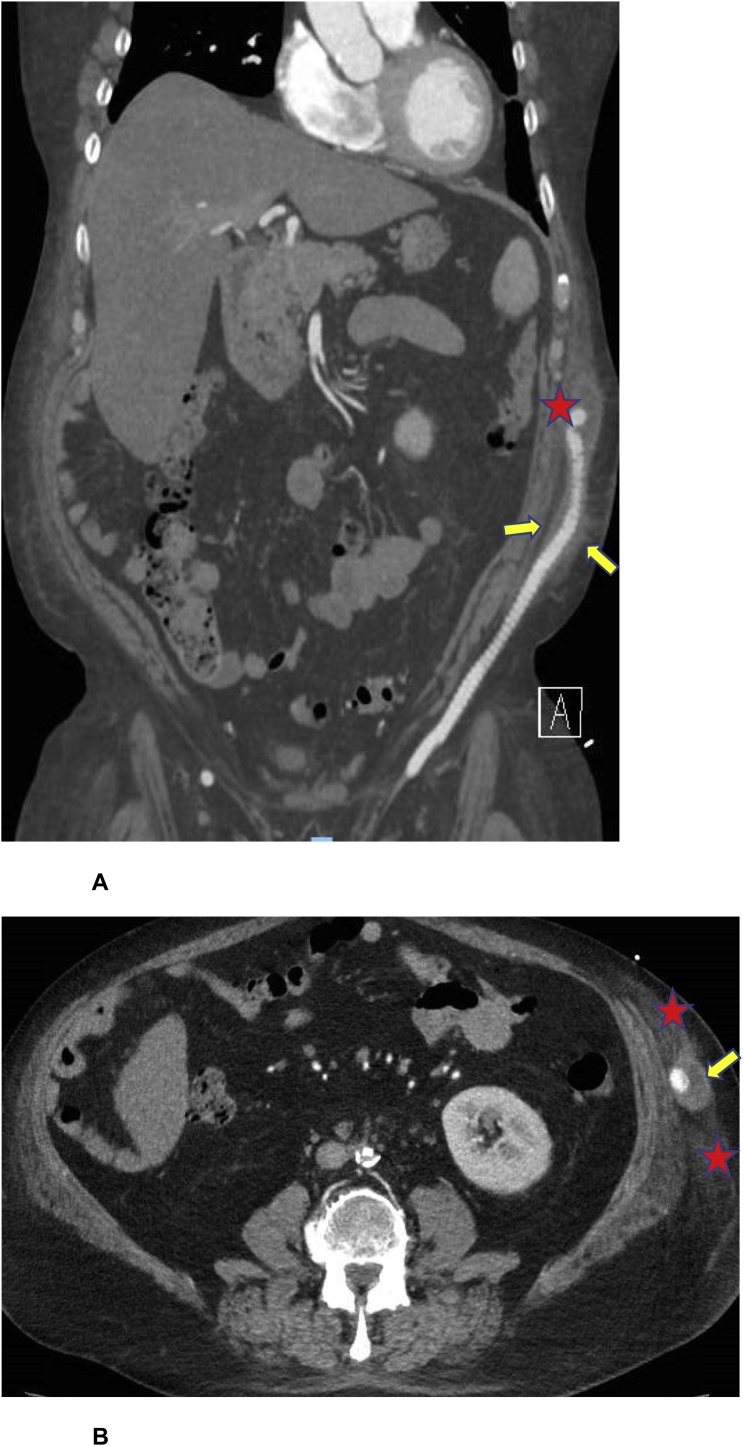
**A.** Computed tomography angiography (*coronal view*) showing a left axillary to femoral artery bypass with fat stranding (*arrows*) and a small surrounding rim enhancing fluid collection (*star*) extending. **B.** Computed tomography angiography (*transversal view*) showing a part of a left axillary to femoral artery bypass with fat stranding (*stars*) and a small surrounding rim enhancing fluid collection (*arrow*).

He was referred to the infectious disease clinic for further evaluation. He denied headaches, respiratory symptoms, abdominal pain, skin rash, gastrointestinal symptoms, and urinary symptoms. He lived in a small town in southeast Iowa and he denied recent travel and contact with sick people, including those with tuberculosis. He had no direct animal exposure although there were several farms within a few miles of his house. He worked as an insurance agent and was indoors most of the day. His blood pressure was 121/75 mmHg, his pulse rate was 109 beats per minute, and he was afebrile. Physical examination was unremarkable except for muscular atrophy and decreased strength in both legs. Repeated CBC and CMP tests were all within normal range. Repeated bacterial, fungal, and mycobacterial blood cultures were negative. CT angiography demonstrated a patent left axillary to femoral artery bypass with fat stranding and a small surrounding rim enhancing fluid collection extending from the level of the diaphragm to the iliac crest (Fig. 2**A** and **B**). *C. burnetii* phase I and phase II IgG antibody titers were > 1:32,768. Phase I and phase II IgM antibody titers were 1:64 and 1:32, respectively. A transesophageal echocardiogram did not show evidence of endocarditis. He was diagnosed with *C. burnetii* vascular graft infection and started on oral doxycycline 100 mg twice and hydroxychloroquine 200 mg three times a day. He underwent explantation of the axillary-bifemoral bypass graft with reimplantation of a cryopreserved artery graft. Cultures obtained during the procedure were negative but *C. burnetii* polymerase chain reaction (PCR) performed on explanted vascular graft was positive. Three weeks later, he returned to clinic for new onset of orthopnea and leg edema. Fluid retention, possibly due to hydroxychloroquine, was suspected and his treatment was changed to doxycycline and ciprofloxacin, with a plan to continue treatment for at least 18 months.

## Discussion

Q fever, a zoonotic infection caused by *C. burnetii* [1], is rare in the United States. The incidence of reported acute or chronic Q fever is 0.28 per million/people [2], though this is likely an underestimate [3]. The organism is typically transmitted from sheep, goats, or cattle to humans via contaminated aerosols [4], but a large proportion of patients do not have a history of close exposures to livestock [5]. *C. burnetii* can be transmitted up to 18 km on gale force winds, with the highest infection risk occurring within 5 km of the source [6].

Approximately 60 % of patients with acute Q fever are asymptomatic [7,8]. Symptomatic patients may have mild symptoms, such as self-limited flu-like symptoms, or severe acute disease, such as pneumonia, hepatitis, acute endocarditis, encephalitis, pericarditis, or myocarditis [5,7]. Symptomatic infection is more common among adults than children and among men than women [9]. On average, 1–5% of patients with acute Q fever will develop chronic infection [7,10,11] months to decades after their primary infections. Culture-negative endocarditis (60–78 %) is the most common presentation, followed by vascular infections (7–8 %) of aneurysms or vascular prostheses, most frequently involving the aorta [11,12]. Bone and joint infections, chronic hepatitis [5,7], and psoas abscesses [8] are other less common manifestations of chronic Q fever. Of patients with chronic Q fever, about 50 % develop chronic infection within 3 months of their acute infections, and 75 % within 6 months [13]. Chronic Q fever is most common among patients with risk factors such as valvulopathy, aortic aneurysms, prosthetic vascular grafts, pregnancy, or immunosuppression, including leukemia, cancer, and human immunodeficiency virus infection [7,9,10].

The diagnosis of Q fever is usually made by serologic testing (indirect immunofluorescence) for antibodies to Phase I and II antigens [5,11]. Acute Q fever is diagnosed by a 4-fold increase in anti-phase II IgG or by a single-phase II IgG titer ≥1:128 or elevated phase II IgM in the appropriate clinical setiing. Anti-phase I IgG at titers of ≥1:800 are indicative of chronic Q fever [5,11]. The diagnosis also can be made using molecular methods including 16S ribosomal ribonucleic acid (rRNA) PCR amplification plus sequencing or with a specific *C. burnetii* PCR [5]. Culture is restricted to specialized laboratories [5,11]. 18-FDG-PET-CT has helped identify vascular infections, endocarditis, and other focal *C. burnetii* infections and to assess the response to therapy [11].

The United States Centers for Disease Control and Prevention and the Q fever Working Group recommend doxycycline for 14 days to treat acute infection. They recommend treating patients who have chronic infection with doxycyline and hydroxychloroquine for 18–24 months, depending on the site of infection and the serologic response [5]. They also recommend obtaining serial antibody levels for more than 5 years after treatment for chronic Q fever to monitor for relapses. Surgical interventions, such as graft removal, are an integral part of treatment for vascular infections and are associated with improved outcomes [14]. Chronic *C. burnetii* infection of aneurysms and vascular grafts is associated with significant morbidity and mortality, with a three-year mortality rate of up to 25 % [14].

In conclusion, Q fever should be considered in the differential diagnosis of vascular graft infections, even in patients who do not have direct contact with animals. The diagnosis can be made using a combination of serologic testing and appropriate imaging.

## Declaration of Competing Interest

The authors report no declarations of interest.

## Funding

No funding.

## Ethical approval

A case report is a medical/educational activity that does not meet the definition of “research”, which is: "a systematic investigation, including research development, testing and evaluation, designed to develop or contribute to generalizable knowledge." Therefore, the activity does not have to be reviewed by ethics committee.

## Consent

Written informed consent was obtained from the patient for publication of this case report and accompanying images. A copy of the written consent is available for review by the Editor-in-Chief of this journal on request.

## Authors contribution

Takaaki Kobayashi: Writing-original draft. Fernando Casado: Writing- review & editing. Jason Barker: Writing - review & editing. Loreen Herwaldt: Writing - review & editing.
